# Delivery of intensity-modulated electron therapy by mechanical scanning: An algorithm study

**DOI:** 10.3389/fonc.2022.1063577

**Published:** 2022-11-24

**Authors:** Pan Ma, Yuan Tian, Minghui Li, Chuanmeng Niu, Yuchun Song, Jianrong Dai

**Affiliations:** Department of Radiation Oncology, National Cancer Center/National Clinical Research Center for Cancer/Cancer Hospital, Chinese Academy of Medical Sciences and Peking Union Medical College, Beijing, China

**Keywords:** electron radiotherapy, scanning, optimization, energy composition, mechanical

## Abstract

**Purpose:**

In principle, intensity-modulated electron therapy (IMET) can be delivered through mechanical scanning, with a robotic arm mounting a linac.

**Materials and methods:**

Here is a scanning algorithm to identify the back-and-forth, top-to-bottom (zigzag) pattern scan sequence. The algorithm includes generating beam positions with a uniform resolution according to the applicator size; adopting discrete energies to achieve the depth of 90% dose by compositing energies; selecting energy by locating the target’s distal edge; and employing the energy-by-energy scan strategy for step-and-shoot discrete scanning. After a zigzag scan sequence is obtained, the delivery order of the scan spots is optimized by fast simulated annealing (FSA) to minimize the path length. For algorithm evaluation, scan sequences were generated using the computed tomography data of 10 patients with pancreatic cancer undergoing intraoperative radiotherapy, and the results were compared between the zigzag path and an optimized path. A simple calculation of the treatment delivery time, which comprises the irradiation time, the total robotic arm moving time, the time for energy switch, and the time to stop and restart the beam, was also made.

**Results:**

In these clinical cases, FSA optimization shortened the path lengths by 12%–43%. Assuming the prescribed dose was 15 Gy, machine dose rate was 15 Gy/s, energy switch time was 2 s, stop and restart beam time was 20 ms, and robotic arm move speed was 50 mm/s, the average delivery time was 124±38 s. The largest reduction in path length yielded an approximately 10% reduction in the delivery time, which can be further reduced by increasing the machine dose rate and the robotic arm speed, decreasing the time for energy switch, and/or developing more efficient algorithms.

**Conclusion:**

Mechanically scanning IMET is potentially feasible and worthy of further exploration.

## 1 Introduction

Intensity-modulated electron therapy (IMET) uses multiple electron beams, each of differing energy and intensity patterns, to deliver a dose distribution that conforms the 90% dose surface to the distal surface of the PTV (1). IMET research on fluence and energy modulation has several pioneers (2–7).

IMET was tried to be delivered using X-ray multileaf collimators (MLCs), similar to X-ray MLCs employed in delivering intensity-modulated X-ray therapy (8–11). However, the air gap of X-ray MLCs is too great; thus, adequate conformity could hardly be acquired (12). Some intensity modulation was realized with scanned electron beams (13), but it requires helium in the treatment head to reduce multiple Coulomb scattering caused by air (14). Furthermore, electron MLC (eMLC) was designed (15–20) and made available by a third party (Euromechanics, Schwarzenbruck, Germany). However, this technology has not been widely applied, possibly because of the high cost of an add-on eMLC, need for integration into commercially available treatment planning systems, and low number of patients requiring electron radiotherapy.

Intraoperative electron radiotherapy also uses a newly designed multirobotic arm apparatus, which comprises a main robotic arm mounted a linac for moving the radiation beam and two subrobotic arms for gripping the accessories. By the cooperative operation of multirobotic arms with automatic control technologies, treatment precision can be improved while greatly reducing the workload (21). This kind of apparatus, that is, the robotic linac, could be used not only for intraoperative radiotherapy (IORT) but also for external radiotherapy, such as skin cancer and keloid excision, with the following beneficial effects (1): multirobotic arms could be integrated together into a flexible on-line image-guided radiotherapy equipment (e.g., a main robotic arm mounted a linac, multiple subrobotic arms selectively mounted a ultrasound device or other imaging devices, an end-gripper for gripping treatment accessories [cone or applicator for electron therapy], and/or a beam stopper to attenuate radiation); (2) a uniform coordinate system may be established for all the robotic arms, allowing the main robotic arm to be guided in aligning the linac to the tumor target with high precision according to the images acquired by the image device; (3) the robotic arm, which has six degrees of freedom, can maneuver and point the beam almost anywhere in space; (4) treatment beams in robotic arm linac have no fixed isocenter, thereby not restricted to isocentric geometry and consequently, can be directed independently; (5) after mounting an X-band accelerator (a beam stopper on the opposite side of the source to reduce the shielding requirements for primary radiation), the linac could be lightweight and compact, thereby delicate and suitable for IORT.

In recent years, the researches on “FLASH” radiotherapy have attracted a great attention for the potential electron clinical applications due to a remarkable sparing of normal tissue. A flexible on-line image-guided radiotherapy equipment integrating multirobotic arms could deliver conformal modulated dose distributions with scanning ultra-high dose rate electron beam and substantially further enhance the therapeutic window in radiotherapy.

Currently, the most advanced proton radiotherapy technique is intensity-modulated radiotherapy, with active scanning being the most advanced mode. Considering the extremely large number of proton beams, the large number of selectable energies, and the complexity of calculations, several optimization methods have been established for active scanning paths for proton radiotherapy (22–26). The three-dimensional scanning intensity-modulated radiotherapy technique for protons can be referenced to achieve scanning intensity-modulated electron therapy (sIMET) using a robotic arm mounting a linac.

This study primarily aimed to establish the mechanical sIMET algorithm, and the following three aspects were considered: (1) determining the scan parameters and strategy, (2) optimizing the scan path, and (3) solving equations.

## 2 Methods

### 2.1 Scan parameter and strategy determination

#### 2.1.1 Scan parameters

This study presumed that an electron accelerator with four discrete energies (E1, E2, E3, and E4) is capable of a depth of 90% dose (R_90_) spanning 10–37 mm for a square field with a side length of 5 mm. This devise might also have dynamic, intensity-controlled discrete spot scanning capabilities with perfect positioning accuracy. The beam R_90_ could be varied in 1 mm steps by compositing two discrete energies. The ratio of the two compositing energies was determined by an exhaustive method with 0.5% accuracy, and then a table of R_90_ and energy correspondence was formed; this table was queried during optimization to identify the beam’s energy.

Next, the scan spots and energies were plainly described. On the beam direction determined by the distribution of a tumor at a certain depth, the beam positions were distributed in one plane. A uniform lateral resolution of beam positions was chosen for the entire plane. After the beam positions were placed at these pixels, energy selection started by locating the target’s distal edge. The appropriate energy values (R_90_) were then matched to the distance between the distal edge and the proximal edge.

#### 2.1.2 Scan strategy

After the scan beam size, position, energy, and dose were determined, the scan strategy was implemented, assigning the mode and sequence of scanning. For a single beam position, two energies may be needed to increase the beam R_90_ in 1 mm steps. In other words, two scan spots may be generated for one beam position. Owing to the differences in target depths, differences in electron beam energies for adjacent beam positions also differed, leading to frequent energy switching and ultimately increasing the delivery time. Therefore, the scanning mode was “energy by energy,” where all scan spots are first grouped by energy, with the same energy grouped into one group. The scanning sequence was the path where the scanning order was optimized to shorten the scanning path and delivered by step-and-shoot discrete scanning.

### 2.2 Scan path length minimization

#### 2.2.1 Traveling salesman formulation

Minimizing the scan path length was a variation on the traveling salesman problem (TSP) in combinatorial optimization. In the classic TSP formulation, one has a map of N cities and must travel a round-trip circuit visiting each city exactly once and returning to the first city, finding the least costly route. The TSP is well researched, and numerous algorithms can be used to solve it. This algorithmic problem can now be applied to electron beam scanning sequences. It is slightly modified in this case, given that the scanner is not required to return to the first scan spot before moving to the next energy. The scan path for each is optimized independently from other energies because the time required to switch to the next beam energy (typically 1–2 s) exceeds that for the robotic arm to move to any scan spot in the next energy (21). The scanning time might have a directly proportional relationship to the path length of the scan. By following this simple model, the scan path length can be optimized, and the cost to move from one scan spot to another is then merely the Euclidean distance, which is expressed as follows:


(1) $$f(P)=\sum_{n=1}^{N-1}\sqrt{{\left(x_{n+1}-x_{n}\right)}^{2}+{\left(y_{n+1}-y_{n}\right)}^{2\;}}\;\;$$

where *N* is the number of scan spots, (*x_n,_ y_n_*) represents the Cartesian coordinates of the n-th scan spot in a given energy group, and *P* indicates the total scan path.

#### 2.2.2 Fast simulated annealing (FSA) algorithm

The FSA was adopted to solve the modified TSP, using a modified cooling function in which the temperature decreases faster than by the logarithmic cooling schedule (27). The neighborhood of states was generated according to a Cauchy probability density distribution, which allows a more efficient search of the solution space. The temperature as a function of the iteration number *k* is expressed as follows:


(2) $$T(k)=\frac{T_{0}}{k^{1/N}}$$

where *T*_0_ is the initial temperature, and *N* is the number of scan spots. Typically, *T*_0_ is set large enough to accept almost all transitions in the beginning. The Cauchy distribution that allows for occasional jumps in the solution space and faster convergence to a global minimum is defined as


(3) $$P(x)=\frac{1}{\text{π}(1+x^{2})}$$

### 2.3 Clinical cases

The most important dosimetric feature of an electron beam is its limited range, which can effectively avoid irradiation of organs behind the tumor target. In addition, it is used in IORT for head and neck (28), abdominal (29, 30), breast (31, 32), and sarcoma (33) tumors.

**Figure 1:**
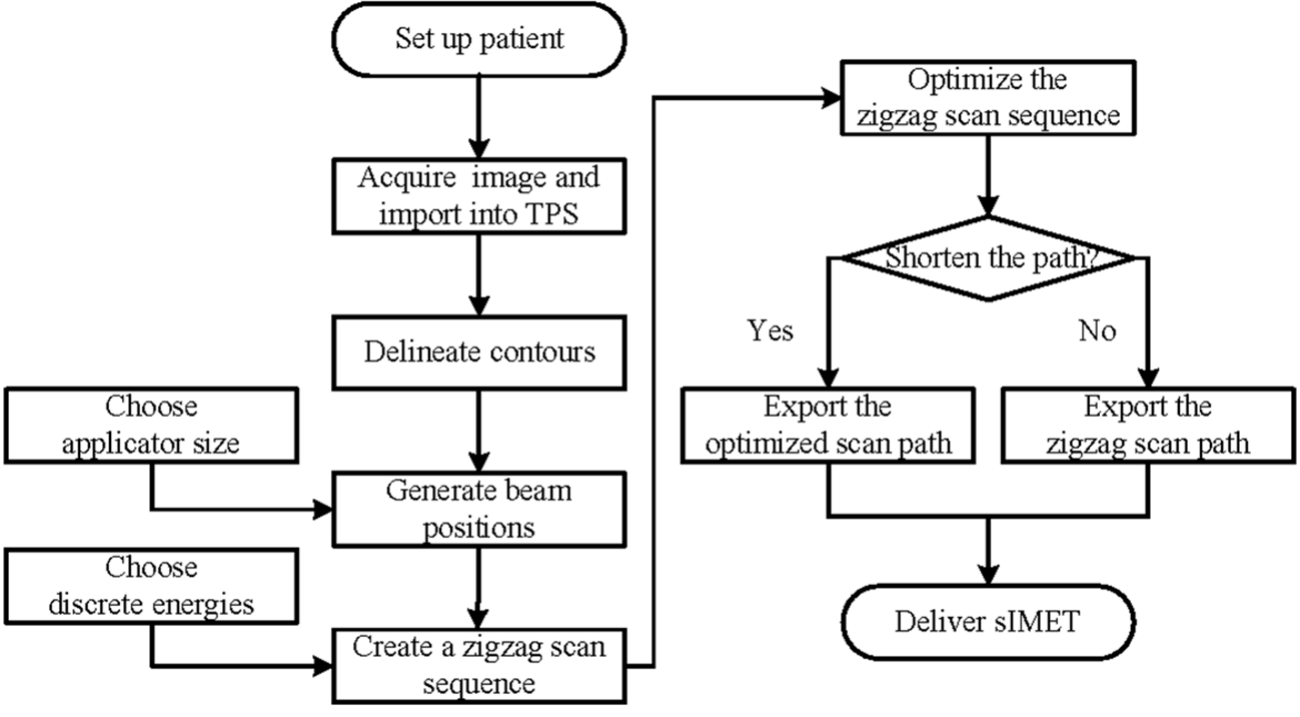
Flow chart of scanning intensity-modulated electron therapy.

The FSA algorithm was evaluated retrospectively using computed tomography (CT) data from 10 patients with pancreatic cancer undergoing IORT with 15 Gy prescription dose at our hospital. A flow chart of the sIMET procedure is shown in **Figure 1**. For IORT, form patient’s setup to completion of irradiation, it will be acceptable to take no more than 20 minutes, including less than 10 minutes for acquisition of images, delineation and planning. The GTV was defined as the lesion visible on preoperative contrast-enhanced CT while the operative recording and additional diagnostic imaging (MR/PET) were considered. PTV resulted from a 5 mm expansion of GTV, with the expansion restricted at anatomical boundaries such as the duodenum, intestine, and colon, around which a 5 mm margin was added. The scan beam size, position, energy, and dose were determined from the PTV and the prescription. Subsequently, the back-and-forth, top-to-bottom (zigzag) pattern scan sequence were identified.

### 2.4 Estimating treatment delivery time

A simple calculation was made on the sIMET for each plan to estimate the treatment delivery time. The delivery time (*T_delivery_*) includes the irradiation time (*T_irradiation_*) from the first scan spot to the last one, the total robotic arm moving time (*T_move_*), the time for switching beam energy from one to the next (*T_energyswitching_*), and the time to stop and restart the electron beam (*T_on/off_*). The time function given by


(4) $$T_{delivery}=T_{irradiation}\;+\;T_{move}\;+\;T_{energyswitching}+\;T_{on/off}$$

where


(5) $$\;T_{irradiation}=\frac{D_{P}}{\dot{D}}\cdot N_{B}$$

(6) $$\;T_{move}=\frac{L}{V_{S}}$$

(7) $$\;T_{energyswitching}=T_{E}\cdot (N_{E}-1)$$

(8) $$\;T_{on/off}=2T_{O}\cdot N_{S}$$

where *D_P_*, $\dot{D}$ , and *N_B_* refer to the prescription dose, dose rate, and the number of beam positions, respectively; *L* and *V_S_* are the path length and the robotic arm’s motion speed, respectively; *T_E_* and *N_E_* denote the time for switching the beam energy from one to the next and the number of energies adopted, respectively; *T_O_* and *N_S_* are the time to stop and restart the electron beam during the discrete scanning and the number of scan spots, respectively.

Taking the initial zigzag path length as a reference, the reduction in treatment delivery time was evaluated due to path length minimization. The motion speed of a realistic robotic arm was 50 mm/s. The robotic arm was stationary at a scan spot until prescription dose delivery, which would take 1 s with a dose rate of 15 Gy/s. The time to prepare a new electron energy was 2 s. The electron beam might need 20 s to stop and restart. This approach suffices to obtain a basic impression of expected relative improvement.

## 3 Results

### 3.1 Results of discrete energy composition

**Figure 2:**
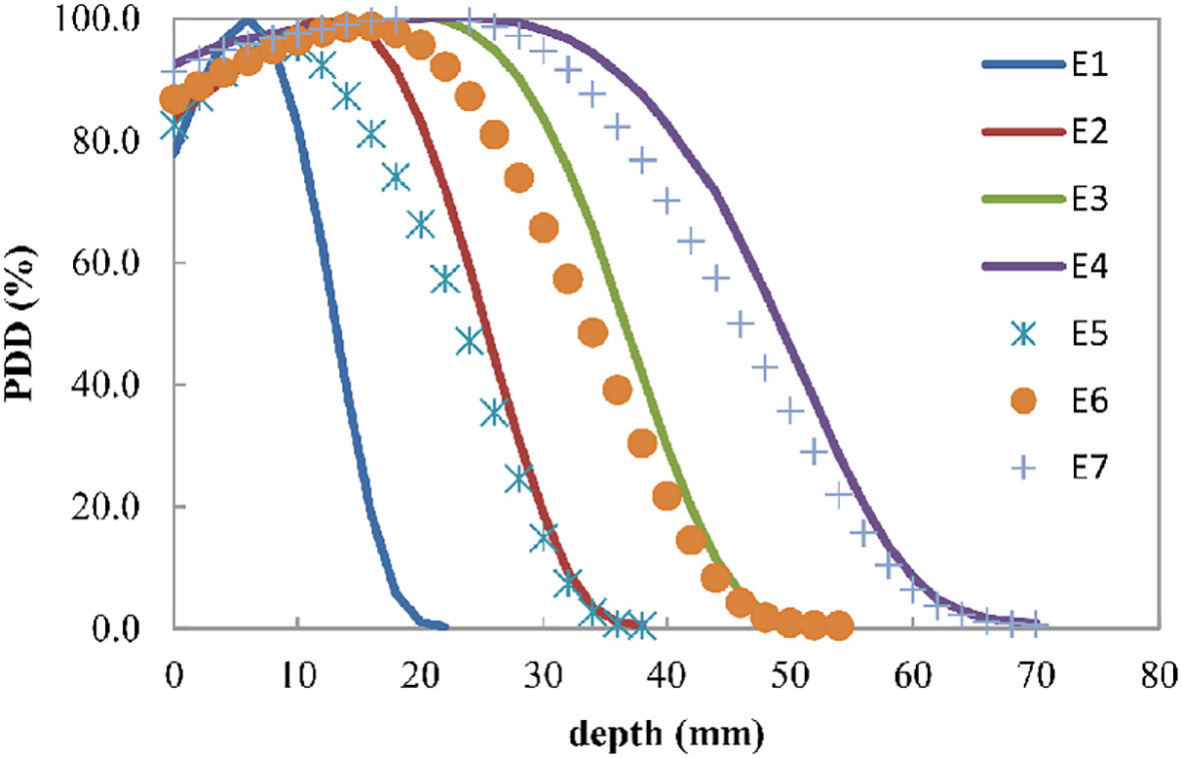
Electron beam percent depth dose curve for the four discrete energies E1, E2, E3, and E4, which can have R_90_ values of 9, 19, 29, and 37 mm, respectively. E5, E6, and E7 are the composited energies, generated by adding 20.5% E1 and 79.5% E2, 27.5% E2 and 72.5% E3, and 23.5% E3 and 76.5% E4, respectively.

**Figure 2** shows the percentage depth dose for four discrete energies and three composited energies. The R_90_ values of E1, E2, E3, and E4 were 9, 19, 29, and 37 mm, respectively. E5, E6, and E7 were composited by 20.5% E1 and 79.5% E2, 27.5% E2 and 72.5% E3, and 23.5% E3 and 76.5% E4, whose R_90_ values were 14, 24, and 33 mm, respectively.

### 3.2 Performance of the FSA algorithm

The computation time for a TSP had become tractable for the optimization of proton therapy scan path (22). In using MATLAB (www.mathworks.com) on a computer with a 3.2 GHz processor, FSA optimization required approximately 82 s for patient 1, comprising 4 energies and 238 scan spots.

#### 3.2.1 Results of scan path length minimization

For the 10 patients, the mean number of scan spots was 18, 39, 55, and 55 for E1, E2, E3, and E4, respectively. Each of these energy groups was optimized independently, with 1 ≤ *N* ≤ 200. After FSA minimization, the change (Δ*S*) in the total path length (*S*) for every patient was reduced by 12.22%–43.07% (**Table 1**).

**Table 1:** Results of the FSA optimization of the total scan path for sIMET plans. *S_i_*, *S_f_*, and Δ*S* refer to the initial, final, and change in path length, respectively.

Patient	Tumor volume(cc)	Number of beam positions	*S_i_* (mm)	*S_f_* (mm)	Δ*S*(%)
1	141.34	159	2462.99	1402.08	-43.07
2	97.94	119	1737.54	1065.31	-38.69
3	100.84	129	1969.26	1306.14	-33.67
4	62.78	93	1344.76	910.32	-32.31
5	62.79	90	1365.73	947.37	-30.63
6	48.11	67	867.6	701.88	-19.1
7	48.13	88	1122.31	909.26	-18.98
8	20.7	42	618.76	526.89	-14.85
9	37.28	75	907.99	774.6	-14.69
10	39.25	75	963.16	845.47	-12.22

**Figure 3:**
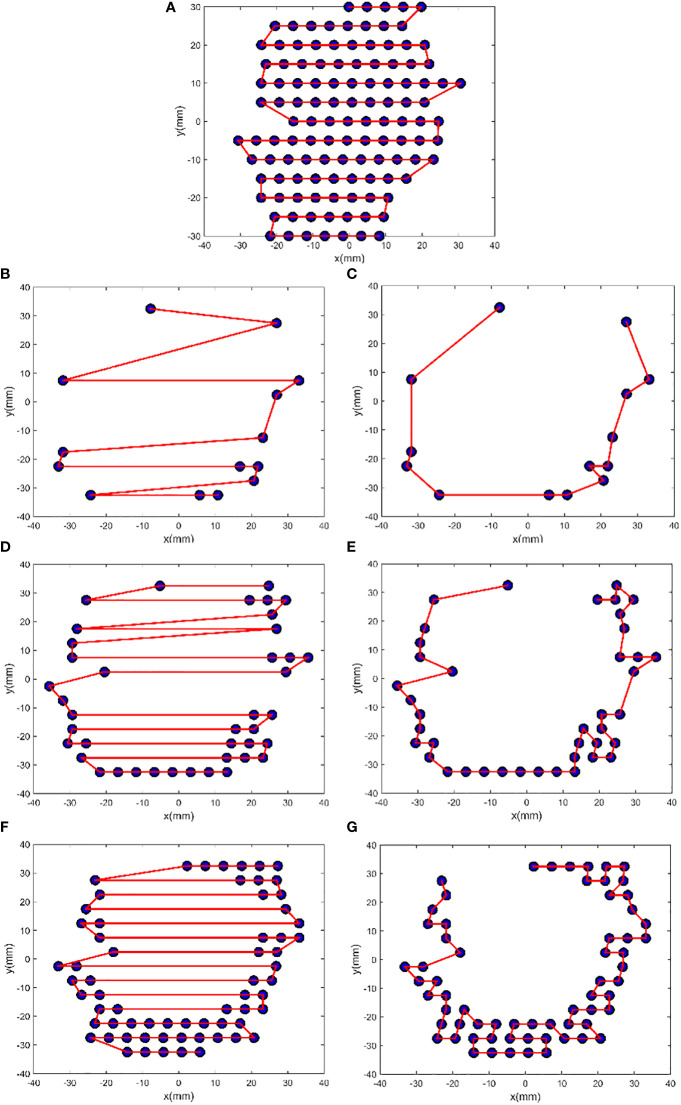
FSA optimization results from patient 1. The plots **(A)**, **(B)**, **(D)**, and **(F)** show the initial zigzag scan path for the energies E4, E1, E2, and E3, and the plots **(C)**, **(E)**, and **(G)** show the FSA solution for E1, E2, and E3, respectively. For E1, *N* = 14, *S_i_* = 386.28 mm, *S_f_* = 190.09 mm, and Δ*S* = −50.79%; for E2, *N* = 41, *S_i_* = 703.00 mm, *S_f_* = 266.70 mm, and Δ*S* = −62.06%; for E3, *N* = 65, *S_i_* = 767.93 mm, *S_f_* = 339.51 mm, and Δ*S* = −55.79%; and for E4, *N* = 118, *S_i_* = 605.78 mm, and Δ*S* = 0.00%.

A larger *N* following FSA could lead to a good improvement; in fact, the largest improvement was found in patient 1 who had the largest *N*. **Figure 3** illustrates the comparison between initial solution and FSA solution from patient 1. For E1, E2, E3, and E4, the path length was reduced by 50.79%, 62.06%, 55.79%, and 0.00%, respectively. Path length reduction was pronounced for sparsely distributed scan spots, whereas for uniform and dense scanning regions, its optimization yielded little or no benefit. Of note, owing to energy composition, one beam position could possibly have two energies; thus, the four energies had 14, 41, 65, and 118 scan spots, for a total of 238, which was more than the number of beam positions (n = 159).

#### 3.2.2 Reduction in treatment delivery time

For the 10 patients, with the mean reduction of 5% due to scan path length optimization, the mean treatment delivery time was 124 ± 38 s, of which the irradiation accounted for 60%. For patient 1, the initial delivery time was 224 s, and the optimized delivery time was 203, of which the irradiation time was 78%.

## 4 Discussion

### 4.1 Path length reduction

In proton radiotherapy, some discontinuities in scanning maps generally result from the inhomogeneity of the patient’s anatomy in the beam line before the target, the use of multiple fields, and the consideration of organs at risk by the optimization process (22). In IORT, few discontinuities could result from the following conditions: only one irradiation field is parallel to the direction of the tumor depth, the tumor is exposed, the organs are pushed away from the irradiation field, and the irradiation field is within 10 cm. The precision in selecting the beam energy, dependent on the tumor depth, determines the number of energy groups. Higher precision indicates more energy, more sparse distribution, and more path length reduction.

### 4.2 Treatment delivery time

Scientists are working on shortening the treatment delivery time because of its multiple benefits, including the increase in the number of patients treated per unit of time, mitigation of patients’ unbearable anxiety, and reduction of the treatment cost. The IORT has very strict total time requirements, and it should ideally be controlled to 15–20 minutes, which is a huge challenge for sIMET. In addition to the treatment delivery time evaluated in this study, extra time for equipment preparation, simulation, planning, and equipment withdrawal are needed.

Using four discrete energies to composite R_90_ with 1 mm accuracy has several advantages in terms of treatment delivery time consumption. First, during the scanning process, switching energy frequently is unnecessary. For patient 1, if the energy increased by 1 for every 1 mm increase in path length, more than 30 energies were needed. Therefore, the time spent on switching energy was more than 60 s. Second, the lower the number of energy, the shorter the path length is. Conversely, the higher the number of energy, the easier it is to form multiple discontinuous scan positions that increase the scanned path length; additionally, the delivery time will still increase even if the path could be shortened by optimization.

There is a difference between the conventionally installed energies and composited energies, and the greater the difference between discrete energies and composited energies, the greater the difference. In this study, only four energies were used for calculation, but are four actually enough? Theoretically, for different tumors, the magnitude and amount of energy used to generate the composited energies should be different, which would be preferably determined using an optimization method. Increasing the number of discrete energies allows for better conformity and more uniform dose and distribution. This approach could be considered if the energy can be switched quickly (e.g., within 50 ms). For patient 1, when the dose rate could be increased to 600 Gy/s (IntraOp Medical Corporation, Sunnyvale, USA), the time after scan path optimization was relatively reduced to 30%. With the 50 mm/s motion speed of a robotic arm, the treatment delivery time could be less than 1 minute.

### 4.3 Intensity modulation and scan beam size

This study revealed that by adjusting the intensity of the scan beam, IMET can be achieved with nonuniform dose distributions according to the intraoperative images such as the CT image (34) and the three-dimensional ultrasound images (35). The electron beam is collimated by the applicator, which determines the scan beam size. Theoretically, the more different the size of the applicators is, the more conformal the dose distributions are for sIMET in the direction perpendicular to the scan beam. However, replacing the applicators with those of various sizes increases the complexity of delivery and prolongs the delivery time. In practice, the selection varies from patient to patient, and a balance between dose conformity and delivery time is needed. Further research is warranted for this direction.

**Figure 4:**
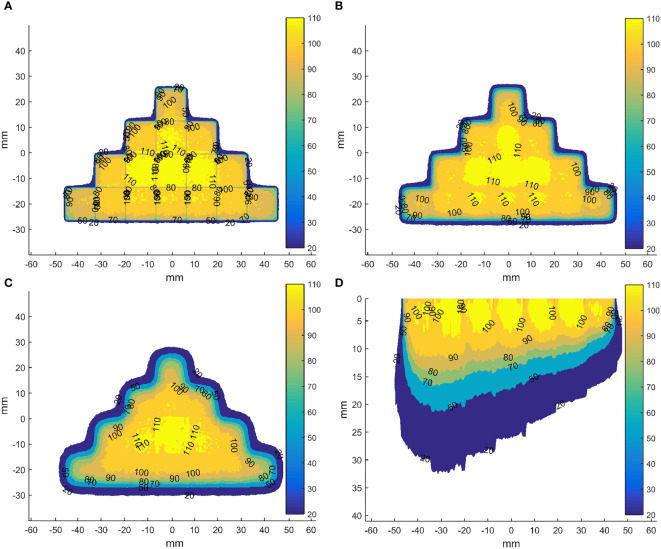
Dose distributions in the coronal planes (perpendicular to the direction of incident electron beam) for the field formed by sixteen abutted square fields of energy 12 MeV with a side length of 5 mm in four rows at depth of 0 **(A)**, 5 **(B)** and 10 mm **(C)** from the phantom surface and in axial plane (parallel to the direction of incident electron beam) through the centre of the field formed by seven abutted square fields with a side length of 5 mm along the negative direction of x-axis, whose R_90_s range from 6 to 12 mm in step of 1 mm by energy composition **(D)**. The dose data has been normalized to the max dose in each square field before abutment.

To explore the dose distributions of the scan beams, we have adopted the validated head of the Mobetron (36) to simulate 3D dose distributions in the homogeneous cubic phantom for a square field with a side length of 5 mm of energies 4, 6, 9 and 12 MeV, whose R_90_ were 6, 8, 10 and 12 mm, respectively. Besides, the 3D dose distributions of two fields formed by sixteen and seven these square fields have been calculated (**Figure 4**). The results show that using the square field can deliver irregular dose distributions, whose boundary is influenced by the side length of square fields. However, we also observed that the dose uniformity deteriorated with increasing depth, which is caused by the inherent dosimetric property of high-energy electron beams that the higher isodose levels tend to show lateral constriction. This might be improved by designing new shaped applicator and adopting repainting scanning method. It should be noted that these dose distributions were not validated due to the conditions.

For sIMET, to ensure the robustness of dose distribution, special considerations are required compared to the processes used for photon. Similar to the unique physical properties of protons, the vulnerability of electrons to uncertainties exists, especially from inter- and intra-fractional variations in anatomy. In addition to anatomy variations, other sources of uncertainty in dose delivered to the patient include the stability of the scan beam, the abutment of the adjacent scan beams. Although the promising results of this algorithm study are encouraging, sIMET may show some limitations. Compared with the conventional IORT, the total treatment deliver time is lengthened due to the addition of simulation, planning, and scan treatment. In addition, due to the scan beam motion, there are organ motion and scanning interactions that need to be considered as well as that the control system and more accurate beam delivery are needed.

These uncertainties and current technological limitations of sIMET may limit the achievement of its true potential. Further study could aim at better understanding the consequences of the various uncertainties on sIMET, reducing the uncertainties and breaking through the technological limitations by image-guidance, adaptive radiotherapy, robust optimization techniques, automatic intelligent planning, ultra-high dose rate, etc. We assert that, with such research, sIMET will be an applied radiotherapy modality in the future.

## 5 Conclusion

This study presents an algorithm that can identify the zigzag pattern scan sequence. The FSA technique is also introduced to optimize the scanning path for mechanical sIMET. Their efficiency has been tested using CT data from 10 patients undergoing IORT for pancreatic cancer. The average delivery time is 124 ± 38 s, which can be further reduced by increasing the machine dose rate and robotic arm speed, decreasing the time for energy switch, and/or developing more efficient algorithms. Mechanically scanning IMET is potentially feasible and worth further exploration.

## Data availability statement

The original contributions presented in the study are included in the article/supplementary material. Further inquiries can be directed to the corresponding author.

## Ethics statement

The studies involving human participants were reviewed and approved by The Clinical Research Committee and the Ethics Committee at the Cancer Hospital, Chinese Academy of Medical Sciences (Approval No. NCC2020C-100). Written informed consent for participation was not required for this study in accordance with the national legislation and the institutional requirements.

## Author contributions

PM and JD contributed to the conception and design of the study. JD provided administrative support. PM, YT, ML, CN and YS provided the materials or patients of the study. PM and JD performed the data analysis and interpretation. PM wrote the first draft of the manuscript. All authors contributed to the article and approved the submitted version.

## Funding

This work was supported by the National Natural Science Foundation of China (82003244), Beijing Municipal Natural Science Foundation (7222314) and National key research and development program of China (2021YFC2400304).

## Acknowledgments

A patent was granted for system and method for planning a scan path for intraoperative radiation therapy in China (patent number 2017103374912).

## Conflict of interest

The authors declare that the research was conducted in the absence of any commercial or financial relationships that could be construed as a potential conflict of interest.

## Publisher’s note

All claims expressed in this article are solely those of the authors and do not necessarily represent those of their affiliated organizations, or those of the publisher, the editors and the reviewers. Any product that may be evaluated in this article, or claim that may be made by its manufacturer, is not guaranteed or endorsed by the publisher.
